# Current Studies and Future Directions of Exercise Therapy for Muscle Atrophy Induced by Heart Failure

**DOI:** 10.3389/fcvm.2020.593429

**Published:** 2020-10-23

**Authors:** Qi Liu, Juan Gao, Jiali Deng, Junjie Xiao

**Affiliations:** ^1^Cardiac Regeneration and Ageing Lab, Institute of Cardiovascular Sciences, School of Life Science, Shanghai University, Shanghai, China; ^2^School of Medicine, Shanghai University, Shanghai, China

**Keywords:** exercise, muscle, atrophy, therapy, targets

## Abstract

Muscle atrophy is a common complication of heart failure. At present, there is no specific treatment to reverse the course of muscle atrophy. Exercise training, due to the safety and easy operation, is a recommended therapy for muscle atrophy induced by heart failure. However, the patients with muscle atrophy are weak in mobility and may not be able to train for a long time. Therefore, it is necessary to explore novel targets of exercise protection for muscle atrophy, so as to improve the quality of life and survival rate of patients with muscular atrophy induced by heart failure. This article aims to review latest studies, summarize the evidence and limitations, and provide a glimpse into the future of exercise for the treatment of muscle atrophy induced by heart failure. We wish to highlight some important findings about the essential roles of exercise sensors in muscle atrophy induced by heart failure, which might be helpful for searching potential therapeutic targets for muscle wasting induced by heart failure.

## Introduction

Muscle atrophy induced by various factors such as heart failure, is a neuropathy with motor and sensory disorders and caused by synthesis inferior to degradation (1). The first study to evaluate the link between muscle wasting and chronic heart failure in patients showed that about 20% patients with clinically heart failure presented with muscle wasting, exhibiting reduced exercise capacity and reduced left ventricular ejection fraction (2). In the elder patients with chronic heart failure, about 20% prevalence showed loss of muscle mass and muscle function compared with healthy elderly people (3). The main clinical manifestations of muscle atrophy were chronic progressive distal limb myasthenia and atrophy, hypoesthesia and disappearance of tendon reflex, accompanied by skeletal deformities such as high arch foot and scoliosis (4, 5). Ubiquitin proteasome and autophagy, two most important cellular proteolysis systems, regulate muscle atrophy by controlling protein turnover in muscle (6–8). In the two systems, four major signaling pathways (IGF1-AKT-FOXO, myostatin, NF-κB, and glucocorticoids) coordinate protein synthesis and degradation simultaneously in muscle atrophy (9–12).

At present, there is no effective treatment to reverse the course of muscle atrophy induced by heart failure. In order to maximize the recovery of independent activity, improve the quality of life and reduce the occurrence of disability as far as possible, symptomatic supportive treatments including drug, surgical operation and rehabilitation training are mainly recommended for therapy (13–15). It has been found that ascorbic acid can reduce the expression of peripheral myelin protein 22 (PMP22) and significantly improve muscle atrophy. However, it failed to show significant effectiveness in various randomized controlled trials in children and adults with muscle atrophy (16, 17). Surgery is the main choice to correct foot deformities caused by muscle atrophy, especially in the late stage. However, there is still insufficient evidence for the long-term impact on the functional recovery of patients (18). Rehabilitation treatment plays a major role in the management of muscle atrophy, especially exercise therapy, which can encourage patients to move, improve blood circulation, prevent contracture, strengthen unaffected muscle strength, improve gait, improve walking ability and quality of life (19).

Here, we summarize different exercise patterns, known targets of exercise and provide a glimpse into the future of exercise for the treatment of muscle atrophy induced by heart failure. We wish to highlight some important findings about the essential roles of exercise sensors in muscle atrophy induced by heart failure, which might be helpful for searching potential therapeutic targets.

## Different Exercise Patterns for the Treatment of Muscular Atrophy

**Figure 1:**
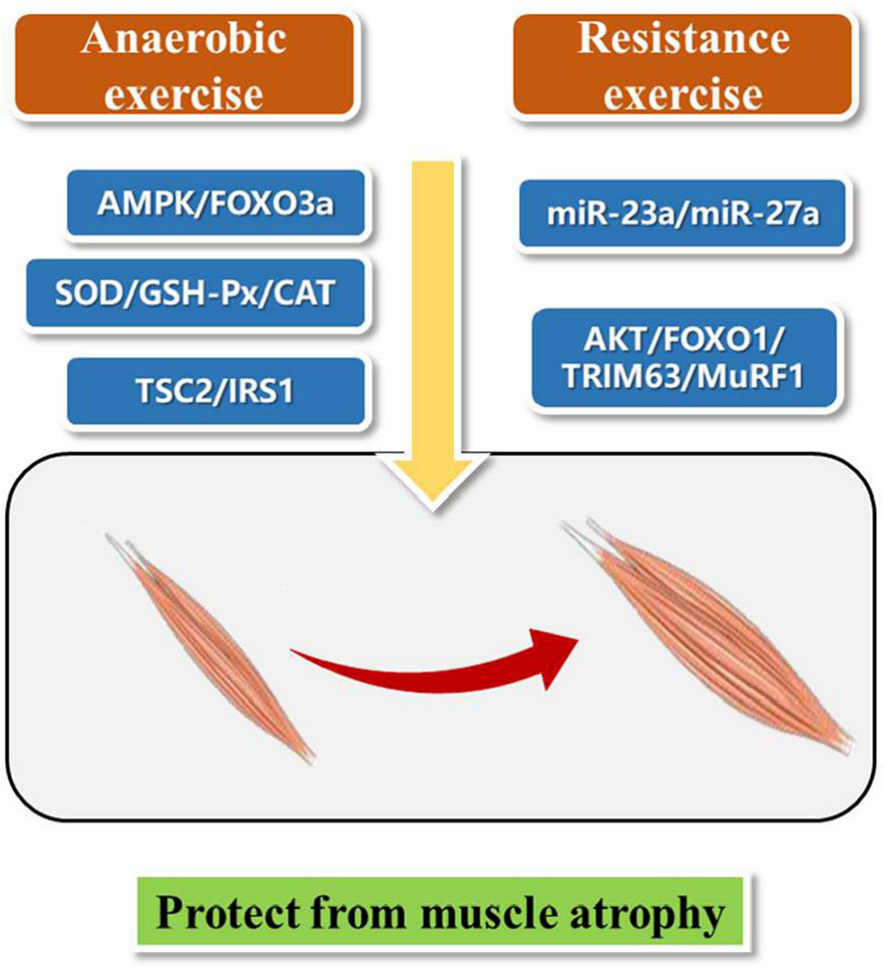
Different exercise patterns for the treatment of muscular atrophy.

Exercise therapy, leading to increase muscle strength, improve aerobic exercise ability, and protect joints from contracture, is mainly divided into aerobic exercise training and anaerobic exercise training according to whether there is sufficient oxygen supply in the body during exercise (20). At the same time, considering the intensity, duration, frequency, location and activity type, exercise can be divided into endurance and resistance exercise (21). Here, we present aerobic exercise and resistance exercise for the treatment of muscle wasting (Figure 1). We hope to find inspiration from these different exercise patterns and bring new ideas for the future research on exercise protection against muscle atrophy.

Studies have shown that aerobic exercise can improve cardiopulmonary function, muscle strength, and activities of daily living (22, 23). A 24-weeks intermittent training (riding training) clinical trial on patients with muscle atrophy showed that intermittent aerobic exercise can improve muscle strength, motor function and subjective feeling of pain and fatigue of patients with muscle atrophy (24). Eight patients with muscle atrophy underwent treadmill and stretching exercise training for 6 months combined with cardiopulmonary function and proprioception rehabilitation training. The results showed that all participants had increased ankle range of motion and 6-min walking test step length (25). Aerobic exercise improves AMPK and skeletal muscle atrophy (26). Activation of AMPK/FOXO3a signaling pathway leads to the activation of protein degradation system and the loss of skeletal muscle mass. Exercise can inhibit the expression of FOXO3a and its downstream targets, and reduce the activity of protein degradation system, thus promoting the recovery of muscular atrophy (27).

Resistance training is the movement against resistance, such as weightlifting (28). Thirty-two patients with muscle atrophy were randomly divided into resistance training group (18 cases) and control group (14 cases) and a single blind crossover design was used to study a period of 16 weeks. Training group showed significantly increase muscle strength of the left hip flexor muscle, improved hip strength, and no negative effect of exercise (29). Another study showed that resistance training can improve the muscle mass, bone mineral density and peripheral muscle volume of heart transplant patients, so as to prevent and reverse muscle atrophy in heart transplantation patients (30).

## Classical Pathways of Exercise Protection for Muscular Atrophy Induced by Heart Failure

**Figure 2:**
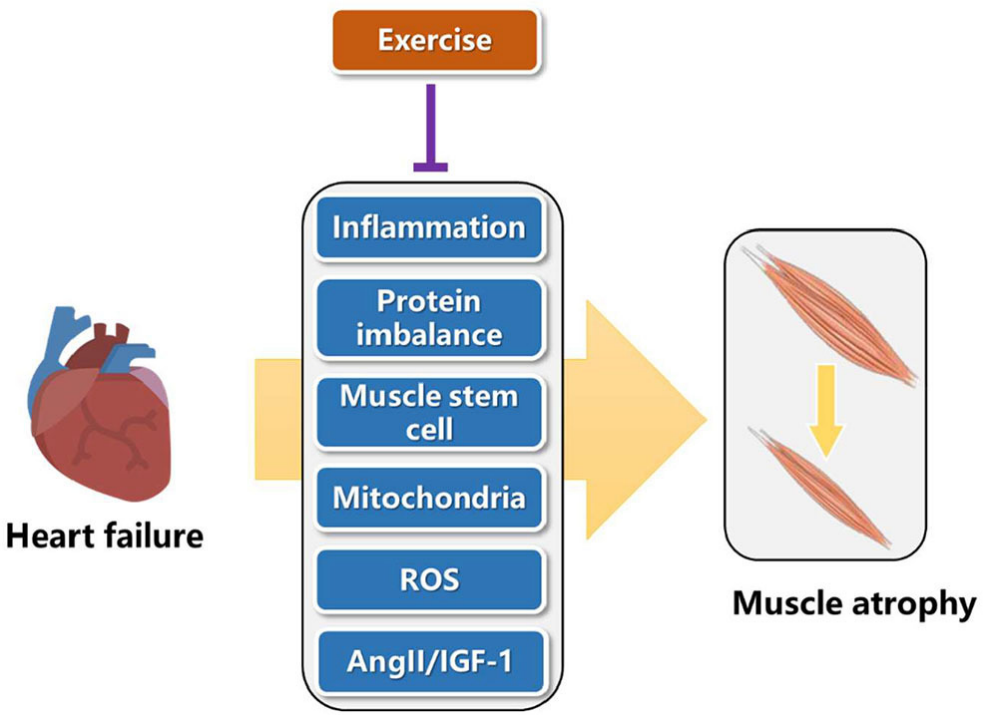
Classical pathways of exercise protection for muscular atrophy induced by heart failure.

Heart failure can increase inflammation, cause lower protein synthesis than degradation, reduce mitochondrial function, increase reactive oxygen species (ROS), change hormone content such as angiotensin II (AngII) and insulin like growth factor 1 (IGF-1), thus leading to muscle atrophy (31). Here, we present that exercise can reverse the adverse pathways caused by heart failure, so as to alleviate muscle atrophy (Figure 2). In the future, these pathways are good directions to explore novel targets of exercise protection for muscular atrophy.

Chronic inflammation is one of the characteristics of muscle atrophy induced by heart failure (31). Exercise decreases the secretion of inflammatory factors such as TNF-α, IL-6, and C-reactive protein (CRP), which can directly reduce the process of protein hydrolysis and catabolism, reduce the degree of muscle loss, so as to achieve the anti-inflammatory effect of exercise (32, 33).

Muscle atrophy is result from muscle protein loss, caused by more muscle protein decomposition than synthesis. In clinical trials, resistance exercise can increase muscle protein synthesis, reverse skeletal muscle consumption, and increase muscle strength and lean weight mass (34). Exercise can accelerate the activity of amino acid transporter and then quickly and efficiently transport amino acid in plasma into muscle cells through transmembrane transporter, promote muscle protein synthesis, reduce the content of free amino acid in plasma, and upregulate the energy regulation and cell nutrition induction. In addition, exercise promotes muscle protein synthesis, related to the activation of mTOR/P70S6K pathway through regulating the expression of myosin heavy chain (MHC) II, which can improve the protein synthesis of fast muscle in skeletal muscle, and increase the volume and mass of muscle (35). Furthermore, exercise can regulate the negative tension of heart failure patients. Under the intervention of exercise, the appetite of patients is improved, and protein intake is enhanced, which also has a certain role in promoting the maintenance of total muscle protein (36).

Muscle stem cells, also known as satellite cells, mainly function to repair damaged muscle tissue (37). Stem cells are normally in a static state and distributed along muscle fibers. When muscle tissue is damaged, they are awakened and activated and self-proliferate and differentiate into myoblasts. On this basis, myoblasts fuse with each other and gradually become polynuclear muscle fibers, realizing the replacement or repair of damaged muscle tissue, so as to restore or increase the function of muscle tissue (38). Exercise intervention experiment found that after proper aerobic exercise, the number and activity of muscle stem cells were significantly increased, and were not affected by the age of the intervener, which has a positive role in repairing damaged muscle cells and alleviating muscle atrophy (39).

Mitochondria function is impaired by heart failure (40, 41). Exercise can promote the rearrangement of mitochondria in skeletal muscle and promote mitochondria metabolism (42). In addition, exercise can maintain the dynamic balance of mitochondria, improve the quality of mitochondria, increase the number of mitochondria and improve the myosin heavy chain (MHC), leading to the enhancement of muscle strength, the improvement of the body's movement ability and the positive promotion effect (43). Physical exercise could activate autophagy in skeletal muscles accompanying with clearance of damaged cell components and dysfunctional mitochondria, which is crucial for muscle homeostasis (44). Further work extended these observations and demonstrated that exercise-induced autophagy plays an important and previously unrecognized role in muscle metabolism (45). These findings provide a mechanism for explaining the well-known beneficial effects of physical activity in healthy individuals.

Studies have pointed out that heart failure reduces the activity of superoxide dismutase and limits the free radical scavenging system (46). Free radicals may cause structural damage of muscle protein by destroying the normal structure of muscle protein molecule, activate ubiquitin proteasome to recognize the damaged muscle protein, accelerate the degradation of muscle protein and promote muscle atrophy (47). Aerobic exercise can improve the activity of superoxide dismutase (SOD) and reduce the level of free radicals. Four weeks of treadmill exercise in mice enhanced the expression of SOD in gastrocnemius mitochondria, inhibited the chain reaction of free radical production, reduced the production of free radicals, and avoided muscle cell damage caused by imbalance of cell oxidative stress level (48). In clinical human experiments, it is found that proper aerobic exercise can improve the activities of SOD, glutathione peroxidase 1 (GSH-Px) and catalase (CAT), and accelerate the speed and ability of the body to remove free radicals and peroxide products (49).

Heart failure decreased IGF-1 and increased AngII (50, 51). Insulin dominated muscle protein synthesis hormone mainly plays a role in skeletal muscle regeneration and repair, which is mainly reflected in that IGF-1 can inhibit muscle protein decomposition, enhance amino acid transport, accelerate nuclear replication process, promote DNA and RNA production, act on ribosomes, accelerate translation process, and promote muscle protein synthesis. Once the plasma insulin concentration or insulin sensitivity is reduced, it will prevent the body's absorption of amino acids and inhibit the synthesis of muscle protein. Studies have found that appropriate aerobic exercise intervention can improve the expression of TSC2 in muscle cells, inhibit the phosphorylation activity of insulin receptor substrates IRS1-Ser307 and IRS1-Ser636/639, enhance the sensitivity of insulin signal pathway, improve the level and sensitivity of insulin in plasma, strengthen the utilization of glucose *in vivo*, promote the synthesis of muscle protein, and inhibit muscle atrophy (52). Patients with congestive heart failure elevated AngII serum concentrations and activate the PKD1/HDAC5/TFEB/MuRF1 pathway to induce skeletal muscle wasting (1). MuRF-1, a component of the ubiquitin-proteasome system involved in muscle proteolysis, is increased in the skeletal muscle of patients with heart failure. Exercise training results in reduced MuRF-1 levels, suggesting that it blocks ubiquitin-proteasome system activation (53).

## Roles of Microrna in Muscle Atrophy Induced by Heart Failure

**Figure 3:**
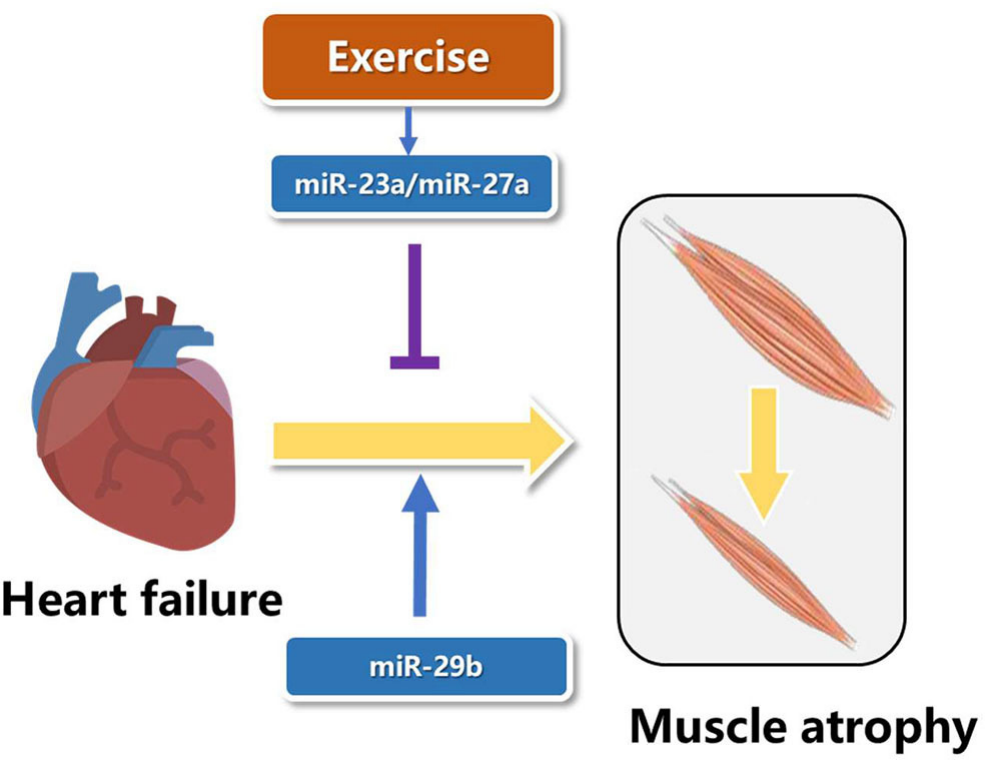
Roles of microRNA in muscle atrophy induced by heart failure.

MicroRNAs are short with a length of about 18–25 nucleotides and non-coding RNAs, which function as inhibition of post-transcription of mRNA's translation (54). Two microRNAs from the opposite side of the pre-microRNA name−3p or−5p (55). In recent years, studies have showed that microRNAs play essential roles in the pathogenesis of muscle atrophy induced by heart failure (56). The expression of miR-23a in muscle atrophy was less than controls, and resistance exercise increased the levels of miR-23a and miR-27a. Overexpression of miR-23a/miR-27a attenuated muscle loss, improved grip strength, activated AKT/FOXO1/TRIM63/MuRF1 pathway, reduced myostatin expression and increased the expression of markers of muscle regeneration (57). Although the role of microRNAs in specific types of muscle atrophy has been reported, it is not clear whether there is a common miRNA target in a variety of muscle atrophy. MiR-29b was consistently up-regulated in muscle atrophy induced by various factors, including Angll, aging, denervation, starvation, dexamethasone treatment, and tumor cachexia. AngII and aging are important factors leading to heart failure (58, 59). Overexpression of miR-29b is sufficient to cause muscle atrophy induced by heart failure, and inhibition of miR-29b can prevent and treat muscle atrophy induced by heart failure (Figure 3). Meanwhile, IGF-1 and PI3K were identified as two target genes of miR-29b. MiR-29b mainly induced muscle atrophy by inhibiting IGF1-AKT-PI3K-mTOR pathway (60, 61). Cardiac cachexia is a common complication of heart failure associated with muscle wasting. MiR-29a-3p, miR-29b-3p, miR-210-5p, miR-214, and miR-489 were decreased in muscle wasting during cardiac cachexia (62). In future research, we wish not only to find more microRNAs involved in exercise protection against muscle atrophy induced by heart failure, but also to use these microRNAs in gene therapy and drug development.

## The Future Directions of Exercise Protecting Muscle Atrophy Induced by Heart Failure

Muscle atrophy is one of the important characteristics of heart failure. Limb motor dysfunction seriously affects the activities of daily living and quality of life of patients, which is an urgent rehabilitation problem to be solved (63). Inhibition of muscle atrophy is of great significance to maintain the body function of patients, enhance the ability of autonomous exercise, relieve pain and fatigue, improve the quality of life of patients and prolong the survival period. There is no intervention found to totally cure muscle atrophy. Rehabilitation therapy is an important pillar of the treatment of the disease. Exercise therapy can improve the motor function of the upper and lower limbs and improve the quality of life. According to the different clinical symptoms of muscle atrophy, how to select the most appropriate exercise therapy, and how to choose the appropriate orthosis support treatment, in order to achieve symptomatic support treatment and function maintenance, are worth further clinical research in the future. Exercise can alleviate muscle atrophy to a certain extent, and the related mechanism may be related to reducing the inflammatory reaction of patients, promoting the synthesis of muscle protein, increasing the activity of muscle stem cells, improving the functional structure of muscle mitochondria, reducing the degree of fatigue, regulating the level of free radicals and hormones, and improving the quality of life of patients. However, the specific mechanism of exercise intervention in muscular atrophy still needs further study.

Although exercise training is recommended for treating muscle wasting induced by heart failure, the patients with muscle atrophy are weak in mobility and may not be able to train for a long time. Patients with heart failure may have intolerance to exercise. For example, heart failure with preserved ejection fraction (HFpEF) is characterized by exercise intolerance (64, 65). For this part of patients, it is a problem that how to safely and effectively perform exercise therapy. Therefore, it is necessary to explore novel targets of exercise protection for muscle atrophy, so as to improve the quality of life and survival rate of patients with muscular atrophy. Further researches are needed to investigate effective targets and treatment according to exercise in muscle atrophy induced by heart failure. We hope to find inspiration from these different exercise patterns, pathways and microRNAs introduced ahead and bring new ideas for the future research on exercise protection against muscle atrophy. Last but not least, we wish to use these targets in gene therapy and drug development in the future.

## Author Contributions

QL wrote the draft and did the figures. JG contributed to the helpful discussion. JX and JD gave the key guidance and edited the draft. All authors read and approved the manuscript.

## Conflict of Interest

The authors declare that the research was conducted in the absence of any commercial or financial relationships that could be construed as a potential conflict of interest.
